# Risk Factors for Ventricular Septal Defects in Murmansk County, Russia: A Registry-Based Study

**DOI:** 10.3390/ijerph15071320

**Published:** 2018-06-24

**Authors:** Anton A. Kovalenko, Erik Eik Anda, Jon Øyvind Odland, Evert Nieboer, Tormod Brenn, Alexandra Krettek

**Affiliations:** 1 Department of Community Medicine, UiT The Arctic University of Norway, 9037 Tromsø, Norway; erik.anda@uit.no (E.E.A.); jon.oyvind.odland@uit.no (J.Ø.O.); tormod.brenn@uit.no (T.B.); alexandra.krettek@uit.no (A.K.); 2 International School of Public Health, Northern State Medical University, 163000 Arkhangelsk, Russia; 3 Department of Biochemistry and Biomedical Sciences, McMaster University, Hamilton, L9H 6C6 ON, Canada; nieboere@mcmaster.ca; 4 Department of Biomedicine and Public Health, School of Health and Education, University of Skövde, 54128 Skövde, Sweden; 5 Department of Internal Medicine and Clinical Nutrition, Institute of Medicine, Sahlgrenska Academy at University of Gothenburg, 41390 Gothenburg, Sweden

**Keywords:** registry, risk factors, ventricular septal defects

## Abstract

Cardiovascular malformations are one of the most common birth defects among newborns and constitute a leading cause of perinatal and infant mortality. Although some risk factors are recognized, the causes of cardiovascular malformations (CVMs) remain largely unknown. In this study, we aim to identify risk factors for ventricular septal defects (VSDs) in Northwest Russia. The study population included singleton births registered in the Murmansk County Birth Registry (MCBR) between 1 January 2006 and 31 December 2011. Infants with a diagnosis of VSD in the MCBR and/or in the Murmansk Regional Congenital Defects Registry (up to two years post-delivery) constituted the study sample. Among the 52,253 infants born during the study period there were 744 cases of septal heart defects (SHDs), which corresponds to a prevalence of 14.2 [95% confidence interval (CI) of 13.2–15.3] per 1000 infants. Logistic regression analyses were carried out to identify VSD risk factors. Increased risk of VSDs was observed among infants born to mothers who abused alcohol [OR = 4.83; 95% CI 1.88–12.41], or smoked during pregnancy [OR = 1.35; 95% CI 1.02–1.80]. Maternal diabetes mellitus was also a significant risk factor [OR = 8.72; 95% CI 3.16–24.07], while maternal age, body mass index, folic acid and multivitamin intake were not associated with increased risk. Overall risks of VSDs for male babies were lower [OR = 0.67; 95% CI 0.52–0.88].

## 1. Introduction

Cardiovascular malformations (CVMs) constitute one of the most common birth defects in newborns [1], and are a leading cause of perinatal and infant mortality [2]. The prevalence of CVMs ranges from three to 12 per 1000 infants and depends on case ascertainment, inclusion criteria, and duration of post birth follow-up [3,4,5]. The etiology of most CVMs is unknown, but possibly up to 30% are attributable to modifiable factors [6]. Genetic causes are estimated to account for <20% of CVMs [7]. Approximately 5–10% of cases are associated with a chromosomal abnormality, 3–5% are related to defects in single genes, and around 2% to environmental factors. Causes of CVMs can also be multifactorial such as an interaction between several genetic and other factors [8]. At present, there is little information on potentially modifiable risk factors, which has made it difficult to develop population-based CVM prevention strategies [9].

The International Statistical Classification of Diseases and Related Health Problems, 10th Revision (ICD-10) classifies a range of CVMs (ICD-10 codes Q20–Q28). ICD-10 code Q21 represents septal heart defects and includes, among others: atrial (ASD), ventricular, atrioventricular septal (AVSD), tetralogy of Fallot, and aorto-pulmonary defects—conditions that range from relatively mild to fatal. Ventricular septal defects (VSDs) are the most common form of cardiovascular malformations. A VSD can occur as an isolated anomaly or in conjunction with other cardiac malformations and/or genetic conditions [10]. Depending on their location in the interventricular septum, septal defects are described as perimembranous, muscular, subarterial, and inflow [11]. Echocardiography is the main imaging modality for the diagnosis and follow-up of VSDs [12]. Children with a VSD are at risk of endocarditis, pulmonary infection, ventricular arrhythmias, and death from heart failure or pulmonary hypertension [13,14,15].

Risk factors for VSDs and ASDs may differ. For example, the effects of maternal alcohol abuse, being overweight and obese are related to VSDs but not ASDs. Conversely, the influence of maternal body mass index (BMI) is evident for ASDs only [16]. High maternal age (≥35) is one of the maternal characteristics known to associate with the risk of septal heart defects (SHDs), and appears to affect both VSDs and ASDs. Smoking, drug abuse, diabetes mellitus, and some infections during pregnancy also appear to be risk factors [17,18,19].

The European Surveillance of Congenital Anomalies and the International Clearinghouse for Birth Defects Surveillance and Research are well-known birth defect monitoring systems [20,21]. Birth defect surveillance in Russia reflects the principles and experiences of these systems, with certain adaptations to the Russian health care system. The registration of congenital defects in regional registries, such as in the Murmansk Regional Congenital Defects Registry (MRCDR), was implemented in 1998. These registries ideally record all birth defects, but currently only 21 are subject to annual reporting including two types of CVMs, specifically hypoplasia of the left heart (ICD-10 Q23.4) and transposition of great vessels (ICD-10 code Q20.3). By contrast, the Murmansk County Birth Registry (MCBR) records and reports on all types of birth defects.

More than 10,000 babies are born with different types of CVM in Russia annually [22]. In 2014, the Federal Russian Statistics Service (Rosstat) estimated the infant mortality resulting from CVM to be 1.5 per 1000 infants. Up to 75% of Russian babies who need life-saving surgical treatment do not receive it. By contrast, in North America modern surgical techniques allow 96–98% of babies with CVM who receive such treatment to survive and live longer [23]. On the basis of the Rosstat data, the prevalence of CVM in Russia ranges from 2.4 to 14.4 per 1000 infants, depending on the region; in Murmansk County, it was 10.9 per 1000 infants in 2010.

The aim of the current study was to identify maternal risk factors for the most frequent CVM, namely ventricular septal heart defects. Our findings constitute a first report on VSDs in Russia.

## 2. Materials and Methods

### 2.1. Data

The study population consisted of all singleton deliveries registered in the MCBR between 1 January 2006 and 31 December 2011 (*n* = 52,253). We searched for cases of SHD followed by VSD within this population by linking information in the MCBR, and in the MRCDR for up to 2 years after birth. We applied a manual linkage procedure based on the maternal hospital ID number and the birth dates of the mother and child. Detailed description of the MCBR and MRCDR establishment and linkage procedure have been published previously [24]. Twelve cases of SHD registered in the MRCDR were not included in the study cohort because they were born outside Murmansk County, or constituted duplicate entries. Supporting data are available upon request.

### 2.2. Ethical Considerations

The study received approval from by the Regional Health Administration of Murmansk County, as well as by the Ethics Committee of Gynecology–Obstetrician Association Group, Murmansk, Russia, and the Regional Ethics Committee, Tromsø, Norway. Ethical code is (reference number): 2013/2146.

### 2.3. Variables

Information on the infant characteristics: birth weight, sex, and gestational age were extracted from the MCBR, as were the following maternal characteristics at delivery: BMI at the first antenatal visit, smoking, alcohol and drug abuse, folic acid and multivitamin intake during pregnancy, and the occurrence of maternal diabetes mellitus type 1 and 2. Smoking, alcohol and drug abuse refer to any usage during pregnancy and were coded as yes/no.

### 2.4. Statistical Analyses

**Figure 1:**
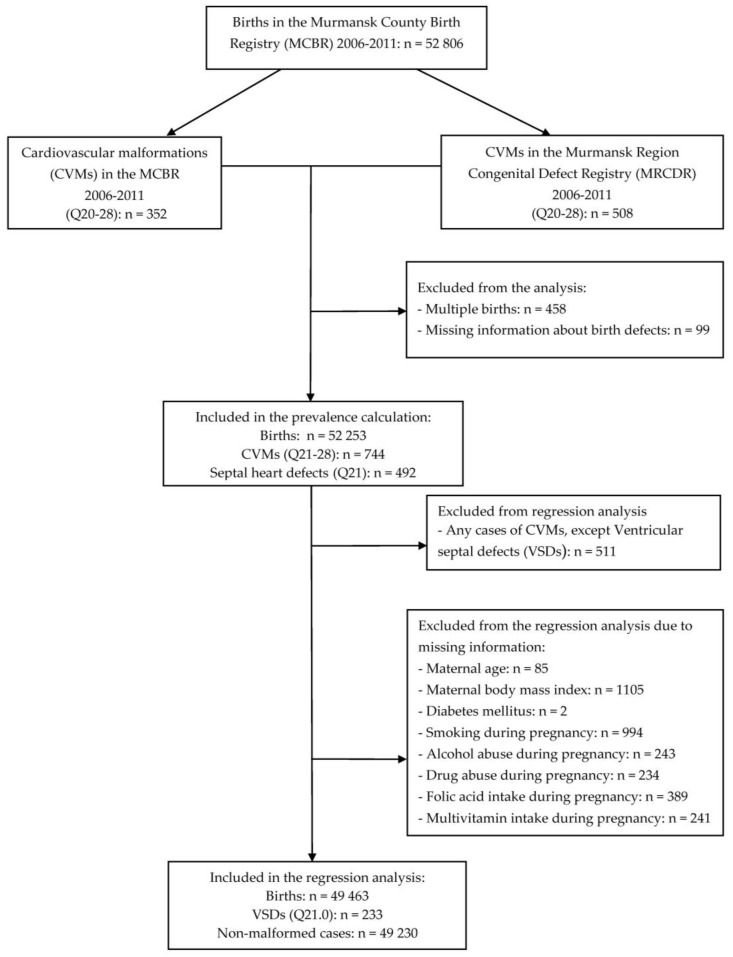
Number of births and exclusions for the combined Murmansk County Birth Registry and the Murmansk Regional Congenital Defects Registry 2006–2011 data. The individual numbers add up to more than the total number excluded because of missing information on two or more variables.

Comparisons of maternal characteristics for groups with VSDs and without any CVMs (control-group) involved chi-square statistics and the two-sample *t*-test for cases and non-cases; the accepted statistical significance level was set at *p* ≤ 0.05. We applied logistic regression to identify factors associated with VSDs. In the latter analysis, the risk and preventive factors considered linked to this birth defect in the literature and those found in the current study to do so. Cases with at least one missing variable were excluded, leaving 49,463 infants for the statistical analyses (Figure 1). A multivariable logistic regression model was used and crude and adjusted odds ratio with 95% confidence intervals (CIs) were calculated. We used the statistical package SPSS v.24.0 (IBM Corp., Armonk, NY, USA, 2016).

## 3. Results

During the study period, 52,253 eligible births were recorded in the MCBR and included 352 cases of CVM; by comparison, 508 CVM cases were noted in the MRCDR. After combining and removing duplicates, 744 cases (ICD-10 code Q20–Q28) remained. The latter correspond to a total prevalence of CVM of 14.2 per 1000. One hundred and sixteen cases of CVM were present in both registries, while 236 appeared only in the MCBR and 392 only in the MRCDR. Isolated SHDs accounted for 492 (66.1%) of all cases of CVM (Table 1).

The subdivision of the observed septal defects by ICD-10 codes was as follows: Q21.0 (VSD) was the most common defect (47.4%), with Q21.1 (ASD; 22.8%) and Q21.9 (unspecified; 23.8%) as secondary major contributors (Table 2).

The mean birth weight (3244.4 g) and gestational age (39.2 weeks) were significantly lower in the group with VSD (Table 3). The proportion of mothers who smoked, abused drugs or abused alcohol during pregnancy was also higher in this group. Multivitamin and folic acid intake were not significantly different in the compared groups.

Although lower birth weight was observed for VSD cases (Table 3), it likely shares a common risk factor with other cardiovascular malformations. For this reason it was not included in the regression analysis. After adjustment, the entry-method regression modelling results (see Table 4) suggest that smoking, alcohol abuse, and maternal diabetes constituted predictors.

Note the significant increases in risk for having a baby with a ventricular septal defect for the following factors were: 8.72 (evidence of maternal diabetes mellitus type 1 and 2); 4.83 (alcohol abuse). Maternal smoking also reached statistical significance (OR = 1.35), while male gender of the baby was protective (OR = 0.67). In a separate multiple logistic regression analysis for ASDs that included the same variables as indicated in Table 4, only male sex of the baby was statistically significant (data not shown).

## 4. Discussion

### 4.1. Selected Risk Factors

#### 4.1.1. Smoking during Pregnancy

Recent epidemiologic studies have demonstrated associations between certain maternal lifestyle factors and the risk of CVMs in offspring including smoking, alcohol abuse, drug abuse, BMI, and psychological factors [9,25,26,27]. Three meta-analyses involving more than 30 studies have investigated the association between maternal smoking during pregnancy and CVMs [28,29,30]. Most feature a positive association between maternal smoking and all CVMs combined. In studies with more detailed analyses, the highest risk (OR = 1.27) occurred for VSDs in a light smokers group [30]. Dose-dependent effects have been reported for atrial septum defects [16]. With reference to Table 4, women who smoked during pregnancy were approximately 35% more likely to have a child with VSD compared with women who did not do so.

Cardiac morphogenesis is complex and risk factors can potentially affect the development of multiple components of the heart. For this reason, the current study focused on ventricular septal defects. The exact mechanisms by which maternal smoking may lead to ventricular septal defects is still unknown. Findings show that maternal smoking has adverse effects on the developing fetus, including hypoxia caused by carbon monoxide, nicotine absorption and toxicity, and reduction in the supply of essential nutrients to the embryonic tissue [30,31,32]. Smoking prevalence is high among women of reproductive age in Russia, which has public health consequences [33]. Even though the adverse effects of smoking on reproductive health are well known, young women continue to smoke, and more than 75% of those who smoked before pregnancy continued to do so throughout their pregnancy [34]. From 2006 to 2011, the prevalence of smoking during pregnancy in Murmansk County increased from 23.8% to 27.9% according to the MCBR statistics.

#### 4.1.2. Sex of the Baby

In our study, male infants were less likely to have VSDs compared with females. Many congenital defects do have a dependence on sex and ethnicity, although no explanation for such deviations has been forthcoming [35]. Within the field of cardiology, the issue of gender differences has received attention because it is recognized that risk factors for cardiovascular defects are unevenly distributed by sex [36]. Possibly, genetic, morphological, and neuro-hormonal factors all contribute towards determining sex-dependent differences in such prevalence [37].

#### 4.1.3. Alcohol Abuse during Pregnancy

Our observation that alcohol abuse during pregnancy is robustly associated with the risk of VSDs is not surprising. Alcohol use by mothers during pregnancy has indeed been observed to associate with different types of CVM in children [38]. The adverse effects of alcohol on the developing fetus comprise a spectrum of structural anomalies and behavioral disabilities and lead to an increased number of newborns with fetal alcohol syndrome [39,40]. The mechanisms by which alcohol consumption during pregnancy results in such heart defects have yet to be determined. In this context, a wide range of teratogenic effects have been documented and suggest that ethanol may produce fetal tissue edema and affect the turgor of the primitive cardiac loop [9]. Furthermore, the signaling systems that allow normal gene activation and cardiogenesis may be affected [41]. Moreover, cell death is an hypothesized mechanism for muscle formation, and alcohol exposure can result in abnormal cell development and cell death [42]. Alcohol-related studies are complicated because of the underreporting of its consumption during pregnancy. Our modelling showed that alcohol-consuming mothers had a 4.83-fold or more increased risk of having a baby with a VSD, although only six mothers of infants with VSDs reported doing so.

#### 4.1.4. Drug Abuse During Pregnancy

Illicit uses by mothers of marijuana, cocaine, heroin or methadone were noted in the MCBR. To date, few studies have addressed drug abuse during pregnancy, and those that do focus on one specific medication or substance. The vasoconstrictors cocaine and marijuana are potential teratogens because exposure to them may result in vascular disruptions and hypoperfusion. A case-control study from Atlanta (USA) showed that maternal cannabis use, according to self- and proxy-reports, was associated with a two-fold increased risk of septal heart defects including VSDs [19]. On the basis of our multivariable logistic regression findings a near two-and-a-half-fold increase in risk was evident for drug abuse, although statistical significance was not reached [OR = 2.39; 95% CI 0.77–7.44].

#### 4.1.5. Diabetes

We found that diabetes mellitus was associated with an eight- to nine-fold increased risk of ventricular septal heart defects in our study. The increasing prevalence of diabetes type 2 among women of childbearing age in Russia makes identifying and implementing effective prevention strategies a high priority [43]. Diabetes mellitus is an important pathogenetic factor that is associated with a wide spectrum of CVMs, including VSDs [12]. Although the mechanisms underlying the association between diabetes and VSDs are not well known, hyperglycemia may play a critical role [44]. Strict glycemic control before conception and during pregnancy appears to reduce risk levels, but achieving and maintaining euglycemia early in pregnancy constitutes a challenge because many diabetic women do not plan their pregnancies [45].

In our study, only diabetes Type 1 and 2 were included. Gestational diabetes, which usually develops at the end of the second trimester was not considered a risk factor because VSDs develop earlier in the pregnancy. Removal of maternal age from the final regression model (see Table 4) changed the OR of diabetes minimally, specifically from 8.72 to 8.93; as did the omission of the BMI variable (to 8.50), and of both BMI and age (8.71). This highlights the importance of diabetes as a risk factor.

#### 4.1.6. Folic Acid and Multivitamins

One of the most important recent discoveries is that periconceptional intake of folic acid may reduce the risk of different types of septal heart defects in offspring, as it does for neural tube defects. This was first identified in an Hungarian study [46]. Findings from subsequent case-control studies have generally been supportive, but not conclusive. In addition, other studies among high-risk groups present ancillary evidence that support a protective effect of folic acid supplements [47,48]. For example, one study showed that women who used medications that are folic acid antagonists exhibited an increased risk of having babies with CVMs, and that this risk was reduced among women who also took multivitamin supplements containing folic acid [49]. In our study, we did not have information on intake of all types of supplements during pregnancy. Nevertheless, in our logistic regression analyses multivitamin and folic acid intake were not associated with any change in the risk of ventricular septal defects.

### 4.2. Strengths and Limitations of the Study

The high quality of the MCBR data is considered a strength of our study [50]. Abortions that occur before 22 weeks of gestation were not included in our study, and this may constitute a limitation in the generalization of our findings. Russia has an active screening regime during pregnancy, and we suspect that some birth defect findings resulted in pregnancy terminations. Our data on smoking, alcohol abuse, and drug abuse are based on clinical evidence and self-reported information and thus may have been underestimated. The power of the current study was limited by the relatively small number of cases and this restricted the number of variables that could be considered in our modelling.

## 5. Conclusions

We showed that alcohol abuse during pregnancy, as well as maternal diabetes mellitus were risk factors for delivering infants with ventricular septal defects. The effects of smoking during pregnancy were marginal. Male offspring were somewhat less susceptible. Potentially numerous cases of VSDs are preventable in Russia if health policy makers were to pay more attention to established risks.

## Figures and Tables

**Table 1:** Incidence and prevalence ^a^ of cardiovascular malformations (CVMs) for the combined data set of newborns registered in the Murmansk County Birth Registry (MCBR) and the Murmansk Regional Congenital Defects Registry (MRCDR) during the period 2006–2011.

ICD-10 Code ^b^	CVM	Cases		Prevalence ^c^
		*n*	%	
Q20	Congenital malformations of cardiac chambers and connections	14	1.9	0.27 (0.2–0.5)
Q21	Septal defects	492	66.1	9.4 (8.6–10.3)
Q22–23	Valves defects	32	4.3	0.6 (0.4–0.9)
Q24	Other congenital malformations of the heart	51	6.9	1.0 (0.8–1.3)
Q25–27	Vessels anomalies	88	11.8	1.7 (1.4–2.1)
Q28	Other congenital malformations of the circulatory system	2	0.3	0.038 (0.037–0.040)
Multiple	Two or more	65	8.7	1.2 (1.0–1.7)
Q20–28	All	744	100	14.2 (13.2–15.3)

^a^ Among all 52,253 study-period newborn. ^b^ ICD-10: International Statistical Classification of Diseases and Related Health Problems, 10th Revision. ^c^ Prevalence per 1000 infants with 95% confidence interval.

**Table 2:** Distribution of septal heart defects (SHDs) for the combined data set of newborns registered in the MCBR and the MRCDR during the period 2006–2011.

ICD-10 Code ^a^	CVM	Cases	
		*n*	%
Q21.0	Ventricular septal defects	233	47.4
Q21.1	Atrial septal defects	112	22.8
Q21.2	Atrio-ventricular septal defects	10	2.0
Q21.3	Tetralogy of Fallot	6	1.2
Q21.4	Aorto-pulmonary septal defects	9	1.8
Q21.8	Other	5	1.0
Q21.9	Unspecified	117	23.8
Q21	All	492	100

^a^ ICD-10: International Statistical Classification of Diseases and Related Health Problems, 10th Revision.

**Table 3:** Characteristics of the groups with ventricular septal defects (VSDs) and those without any CVMs (Q21) for the combined data set of newborns registered in the MCBR and the MRCDR during the period 2006–2011.

Variables	Cases, *n* = 233 ^a^		Non-Cases, *n* = 49,230 ^a^		*p*-Value ^b^
	X	SD or %	X	SD or %	
Infant characteristics					
Birth weight (g), mean ± SD	3244.4	677.6	3377.2	546.5	<0.001
<2500	30	12.9	2211	4.5	
2500–3999	179	76.8	41,958	85.2	
≥4000	24	10.3	5061	10.3	
Sex, male	98	42.1	25,571	52.0	0.003
Maternal characteristics					
Age at delivery (years), mean ± SD	26.06	5.44	26.79	5.27	0.43
<18	3	1.3	727	1.5	
18–35	209	89.7	45,429	92.3	
>35	21	9.0	3074	6.2	
Gestational age (weeks), mean ± SD	39.2	2.3	39.5	2.2	0.05
BMI (kg/cm^2^), mean ± SD	23.37	4.57	23.49	4.28	0.67
<18.5	16	6.9	3103	6.3	
18.5–24.9	157	67.4	32,325	65.7	
25.0–29.9	40	17.2	9712	19.7	
30.0–34.9	15	6.4	3084	6.3	
35.0–39.9	3	1.3	782	1.6	
≥40	2	0.9	224	0.5	
Smoking during pregnancy	74	31.8	12,234	24.9	0.02
Alcohol abuse during pregnancy	6	2.6	178	0.4	<0.001
Drugs abuse during pregnancy	4	1.7	173	0.4	0.01
Folic acid intake during pregnancy	175	75.1	36,545	74.2	0.76
Multivitamins intake during pregnancy	211	90.6	45,568	92.6	0.25
Diabetes mellitus Type 1 or 2	4	1.7	94	0.2	0.001

^a^ The number of cases and non-cases are less than the entire study population due to missing values of the independent variables. ^b^ *t*-test, Chi-square test, or Fisher’s exact test.

**Table 4:** Multivariable logistic regression. Crude and adjusted odds ratio (OR) with 95% confidence interval (CI) of ventricular septal defects ^a^ for the combined data set of newborns registered in the MCBR and the MRCDR during the period 2006–2011.

Variables	Crude		Adjusted ^b^	
	OR	95% CI	OR	95% CI
Maternal age at delivery (years)				
<18	0.90	0.29–2.81	0.84	0.27–2.65
18–35	1	Reference	1	Reference
>35	1.49	0.95–2.33	1.53	0.97–2.41
Maternal BMI (kg/cm^2^) ^c^				
<18.5	1.06	0.63–1.78	1.10	0.66–1.85
18.5–24.9	1	Reference	1	Reference
>25	0.90	0.66–1.21	0.86	0.63–1.16
Smoking during pregnancy	1.41	1.07–1.86	1.35	1.02–1.80
Alcohol abuse during pregnancy	7.28	3.20–16.60	4.83	1.88–12.42
Drugs abuse during pregnancy	4.95	1.82–13.46	2.39	0.77–7.44
Folic acid intake during pregnancy	1.05	0.78–1.41	1.14	0.84–1.55
Multivitamins intake during pregnancy	0.77	0.50–1.20	0.99	0.69–1.43
Diabetes mellitus type 1 or 2	9.13	3.33–25.04	8.72	3.16–24.07
Sex (male)	0.67	0.52–0.87	0.67	0.52–0.88

^a^ There were 233 cases and 49,230 non-cases. ^b^ Each variable is adjusted for the other listed variables. ^c^ BMI: body mass index.

## References

[1] Ou Y., Mai J., Zhuang J., Liu X., Wu Y., Gao X., Nie Z., Qu Y., Chen J., Kielb C. (2016). Risk factors of different congenital heart defects in Guangdong, China. Pediatr. Res..

[2] Jortveit J., Oyen N., Leirgul E., Fomina T., Tell G.S., Vollset S.E., Eskedal L., Dohlen G., Birkeland S., Holmstrom H. (2016). Trends in mortality of congenital heart defects. Congenit. Heart Dis..

[3] Postoev V.A., Talykova L.V., Vaktskjold A. (2014). Epidemiology of cardiovascular malformations among newborns in monchegorsk (North-West Russia): A register-based study. J. Public Health Res..

[4] Pei L., Kang Y., Zhao Y., Yan H. (2017). Prevalence and risk factors of congenital heart defects among live births: A population-based cross-sectional survey in Shaanxi province, Northwestern China. BMC Pediatr..

[5] Loffredo C.A. (2000). Epidemiology of cardiovascular malformations: Prevalence and risk factors. Am. J. Med. Genet..

[6] Wilson P.D., Loffredo C.A., Correa-Villasenor A., Ferencz C. (1998). Attributable fraction for cardiac malformations. Am. J. Epidemiol..

[7] Gelb B.D., Chung W.K. (2014). Complex genetics and the etiology of human congenital heart disease. Cold Spring Harb. Perspect. Med..

[8] Van der Bom T., Zomer A.C., Zwinderman A.H., Meijboom F.J., Bouma B.J., Mulder B.J. (2011). The changing epidemiology of congenital heart disease. Nat. Rev. Cardiol..

[9] Feng Y., Yu D., Yang L., Da M., Wang Z., Lin Y., Ni B., Wang S., Mo X. (2014). Maternal lifestyle factors in pregnancy and congenital heart defects in offspring: Review of the current evidence. Ital. J. Pediatr..

[10] Batra M., Heike C.L., Phillips R.C., Weiss N.S. (2007). Geographic and occupational risk factors for ventricular septal defects: Washington State, 1987–2003. Arch. Pediatr. Adolesc. Med..

[11] Rojas C.A., Jaimes C., Abbara S. (2013). Ventricular septal defects: Embryology and imaging findings. J. Thorac. Imaging.

[12] Minette M.S., Sahn D.J. (2006). Ventricular septal defects. Circulation.

[13] Graham T.P., Gutgesell H.P., Emmanouilides G.C. (1995). Ventricular Septal Defects. Moss and Andrews Heart Disease in Infants, Children and Adolescents: Including the Fetus and Young Adult.

[14] Abdulla R. (1998). Perspective in pediatric cardiology. Volume 5. Genetic and environmental risk factors of major cardiovascular malformations. Pediatr. Cardiol..

[15] Brickner M.E., Hillis L.D., Lange R.A. (2000). Congenital heart disease in adults. Second of two parts. N. Engl. J. Med..

[16] Larkin S.A. (2013). Atrial and Ventricular Septal Defects: Molecular Determinants, Impact of Environmental Factors and Non-Surgical Interventions.

[17] Tikkanen J., Heinonen O.P. (1991). Risk factors for ventricular septal defect in Finland. Public Health.

[18] Tikkanen J., Heinonen O.P. (1992). Risk factors for atrial septal defect. Eur. J. Epidemiol..

[19] Williams L.J., Correa A., Rasmussen S. (2004). Maternal lifestyle factors and risk for ventricular septal defects. Birth Defects Res. A.

[20] Greenlees R., Neville A., Addor M.C., Amar E., Arriola L., Bakker M., Barisic I., Boyd P.A., Calzolari E., Doray B. (2011). Paper 6: EUROCAT member registries: Organization and activities. Birth Defects Res. A.

[21] Botto L.D., Robert-Gnansia E., Siffel C., Harris J., Borman B., Mastroiacovo P. (2006). Fostering international collaboration in birth defects research and prevention: A perspective from the International Clearinghouse for Birth Defects Surveillance and Research. Am. J. Public Health.

[22] Russian Institute of Public Health (2015). Report of Federal Informational Center of Gene Registering and Birth Defects’ Monitoring.

[23] Jacobs J.P., Jacobs M.L., Mavroudis C., Chai P.J., Tchervenkov C.I., Lacour-Gayet F.G., Walters H., Quintessenza J.A. (2010). Atrioventricular septal defects: Lessons learned about patterns of practice and outcomes from the congenital heart surgery database of the society of thoracic surgeons. World J. Pediatr. Congenit. Heart Surg..

[24] Kovalenko A.A., Brenn T., Odland J.O., Nieboer E., Krettek A., Anda E.E. (2017). Under-reporting of major birth defects in northwest russia: A registry-based study. Int. J. Circumpolar Health.

[25] Kuciene R., Dulskiene V. (2008). Selected environmental risk factors and congenital heart defects. Medicina (Kaunas).

[26] Cai G.J., Sun X.X., Zhang L., Hong Q. (2014). Association between maternal body mass index and congenital heart defects in offspring: A systematic review. Am. J. Obstet. Gynecol..

[27] Sullivan P.M., Dervan L.A., Reiger S., Buddhe S., Schwartz S.M. (2015). Risk of congenital heart defects in the offspring of smoking mothers: A population-based study. J. Pediatr..

[28] Hackshaw A., Rodeck C., Boniface S. (2011). Maternal smoking in pregnancy and birth defects: A systematic review based on 173 687 malformed cases and 11.7 million controls. Hum. Reprod. Update.

[29] Kallen K. (1999). Maternal smoking and congenital heart defects. Eur. J. Epidemiol..

[30] Lee L.J., Lupo P.J. (2013). Maternal smoking during pregnancy and the risk of congenital heart defects in offspring: A systematic review and metaanalysis. Pediatr. Cardiol..

[31] Correa A., Levis D.M., Tinker S.C., Cragan J.D. (2015). Maternal cigarette smoking and congenital heart defects. J. Pediatr..

[32] Alverson C.J., Strickland M.J., Gilboa S.M., Correa A. (2011). Maternal smoking and congenital heart defects in the baltimore-washington infant study. Pediatrics.

[33] Kharkova O.A., Grjibovski A.M., Krettek A., Nieboer E., Odland J.O. (2017). Effect of Smoking Behavior before and during Pregnancy on Selected Birth Outcomes among Singleton Full-Term Pregnancy: A Murmansk County Birth Registry Study. Int. J. Environ. Res. Public Health.

[34] Bergmann R.L., Bergmann K.E., Schumann S., Richter R., Dudenhausen J.W. (2008). [Smoking during pregnancy: Rates, trends, risk factors]. Z. Geburtshilfe Neonatol..

[35] Nembhard W.N., Wang T., Loscalzo M.L., Salemi J.L. (2010). Variation in the prevalence of congenital heart defects by maternal race/ethnicity and infant sex. J. Pediatr..

[36] Engelfriet P., Mulder B.J. (2009). Gender differences in adult congenital heart disease. Neth. Heart J..

[37] Mercuro G., Bassareo P.P., Mariucci E., Deidda M., Zedda A.M., Bonvicini M. (2014). Sex differences in congenital heart defects and genetically induced arrhythmias. J. Cardiovasc. Med. (Hagerstown).

[38] Yazici A.B., Uslu Yuvaci H., Yazici E., Halimoglu Caliskan E., Cevrioglu A.S., Erol A. (2016). Smoking, alcohol, and substance use and rates of quitting during pregnancy: Is it hard to quit?. Int. J. Womens Health.

[39] Popova S., Lange S., Probst C., Gmel G., Rehm J. (2017). Estimation of national, regional, and global prevalence of alcohol use during pregnancy and fetal alcohol syndrome: A systematic review and meta-analysis. Lancet Glob. Health.

[40] Tsang T.W., Elliott E.J. (2017). High global prevalence of alcohol use during pregnancy and fetal alcohol syndrome indicates need for urgent action. Lancet Glob. Health.

[41] Shillingford A.J., Weiner S. (2001). Maternal issues affecting the fetus. Clin. Perinatol..

[42] Menegola E., Broccia M.L., Di Renzo F., Giavini E. (2001). Acetaldehyde in vitro exposure and apoptosis: A possible mechanism of teratogenesis. Alcohol.

[43] Dedov I., Maslova O., Suntsov Y., Bolotskaia L., Milenkaia T., Besmertnaia L. (2009). Prevalence of diabetic retinopathy and cataract in adult patients with type 1 and type 2 diabetes in russia. Rev. Diabet. Stud..

[44] Nielsen G.L., Norgard B., Puho E., Rothman K.J., Sorensen H.T., Czeizel A.E. (2005). Risk of specific congenital abnormalities in offspring of women with diabetes. Diabet. Med..

[45] Ray J.G., O’Brien T.E., Chan W.S. (2001). Preconception care and the risk of congenital anomalies in the offspring of women with diabetes mellitus: A meta-analysis. QJM.

[46] Czeizel A.E. (2011). Periconceptional folic acid-containing multivitamin supplementation for the prevention of neural tube defects and cardiovascular malformations. Ann. Nutr. Metab..

[47] Botto L.D., Krikov S., Carmichael S.L., Munger R.G., Shaw G.M., Feldkamp M.L. (2016). National Birth Defects Prevention Study. Lower rate of selected congenital heart defects with better maternal diet quality: A population-based study. Arch. Dis. Child. Fetal Neonatal. Ed..

[48] Botto L.D., Mulinare J., Erickson J.D. (2003). Do multivitamin or folic acid supplements reduce the risk for congenital heart defects? Evidence and gaps. Am. J. Med. Genet. A.

[49] Hernandez-Diaz S., Werler M.M., Walker A.M., Mitchell A.A. (2000). Folic acid antagonists during pregnancy and the risk of birth defects. New Engl. J. Med..

[50] Anda E.E., Nieboer E., Voitov A.V., Kovalenko A.A., Lapina Y.M., Voitova E.A., Kovalenko L.F., Odland J.O. (2008). Implementation, quality control and selected pregnancy outcomes of the murmansk county birth registry in russia. Int. J. Circumpolar Health.

